# Gastric Digestion in Babies

**Published:** 1908-01-11

**Authors:** 


### Diseases of Children.

### GASTRIC DIGESTION IN BABIES.

It is not yet settled whether the lactic acid so constantly found in the infant's stomach is a normal or pathological product. In comparison with adults the gastric juice has a relatively high acidity. Recently Sedgwick has found in the stomach contents a ferment, *lipase*, which splits up milk-fats into free fatty acids and produces high acidity. The secretion of hydrochloric acid is decreased in ill-health and in even slight disturbances of digestion it may be completely suppressed. In the breast-fed babe, free acid appears in about an hour and a quarter after a meal; in the bottle-fed, in two hours. Though present immediately after a meal it is at first combined with the proteid and alkaline salts of the milk, and thus concealed. Because of the greater amount of such substances in cow's milk, free acid takes longer to appear. The combined acid has no bactericidal power, but the free acid, though not definitely bactericidal in the strength met with in the stomach, exercises an inhibitory effect on microbial growth. From this it follows that disinfection of the stomach contents takes place more readily in the breast-fed than in the bottle-fed, for free hydrochloric acid appears in the former at an earlier period after the meal. So, too, it is important that there should be a definite interval between meals, sufficiently long to permit the secretion of free acid, and that this interval should be longer when the diet consists of cow's milk than in the case of human milk. The administration of dilute hydrochloric acid to infants after meals is hereby shown to be based on sound chemical conditions.

Many of the cases of supposed hyperchlorhydria have not been sufficiently well investigated to afford reliable proof of the actual existence of an excess of the acid. A high total acidity is an insufficient test. In certain cases the proof has been sufficiently accurate to show that this hyperacidity can occur in babies. Using dimethylamidoazobenzol as an indicator (Troepfer's method), Knöpfelmacher found it definitely present. Possibly some of the cases of congenital hypertrophic stenosis of the pylorus are secondary to hyperchlorhydria, but further observations are necessary before accepting such a view. Notably it is important to make quantitative estimations of the amount of acid in this affection and in other diseases. Probably hyperchlorhydria does occur in babies and may give rise to pyloric spasm. Assuming this to be true, further proof is requisite to show that the spasm causes hypertrophy. In support of this theory it should be mentioned that Freund claims to have cured congenital stenosis of the pylorus by a diet of undiluted cow's milk. Theoretically, fresh undiluted cow's milk should prove the best diet, because of its higher combining power.

R. Miller and W. H. Willcox (*Lancet*, 1907, vol. ii., p. 1671) report that the gastric secretion varies in three types of cases. In marasmus both acidity and ferment activity are diminished; no mucin is secreted, and there is no retention of food in the stomach. In five cases of hypertrophic stenosis, four verified after death, there were much mucin, food retention, and marked increase in ferment activity. Acidity was variable, tending to be decreased in accordance with the development of secondary gastritis. Milk is clotted firmly and quickly by the gastric juice, and is a bad diet. In pyloric spasm or acid dyspepsia there are retention of food and increased acidity, normal or subnormal ferment activity, and no mucin. A dilated stomach may be seen to "stand up" on tapping.

Deficiency of pepsin is not common in babies, though a few such cases have been described. The rennet enzyme is always present. Its action on cow's milk differs from that on human milk. Fresh, undiluted cow's milk, unshaken, forms a coherent clot. Human milk does not clot at all, unless a little hydrochloric acid is added, and then it only forms a fine, flocculent coagulum. If cow's milk is sufficiently diluted and agitated the clot formed is similar to that with human milk. Biedert states that cow's milk clots in the stomach in the same way as human milk, provided that the ratio of fat to proteid is the same. Dilution with egg-albumin, gelatin solution, or a cereal decoction has a similar effect, dependent on the degree of dilution and reduction of acidity, rather than on the nature of the diluent. It follows, therefore, that heavy lumpy clots will form in the stomach if milk is fresh and undiluted, and if the motor power of the stomach is deficient. An excess of fat in the food has a deleterious effect on proteid digestion, either through its influence on gastric secretion or by mechanically coating the particles of proteid and increasing their resistance to the action of the gastric juice. Large clots are supposed to be bad for the infant by offering resistance to impregnation with the digestive juices and so to peptonisation. Test tube experiments on the artificial digestion of casein show that it takes place as well with large clots as with flocculent ones (Escherich). Some authors have ascribed to rennet the power of dehydrating peptone and converting it into an albumin. The change is said to take place in the mucous membrane of the stomach and intestines, the various products of proteid digestion being converted into a substance, named "plastein," from which proteid tissues are built up. Another view has been enunciated by Rotondi that by the action of the rennet ferment on casein a proteid body is split off, which is soluble in acetic acid and is not coagulated by heat. Further information on these various points will be found in v. Noorden's work on "Metabolism and Practical Medicine."

The general conclusions are that rennet ferment is always present; that pepsin is almost invariably present; that hydrochloric acid is often decreased in amount, sometimes entirely suppressed, and occasionally excessive.

